# Sensory Properties and Acceptability of Fermented Pearl Millet, a Climate-Resistant and Nutritious Grain, Among Consumers in the United States—A Pilot Study

**DOI:** 10.3390/foods14050871

**Published:** 2025-03-03

**Authors:** May M. Cheung, Lauren Miller, Jonathan Deutsch, Rachel Sherman, Solomon H. Katz, Paul M. Wise

**Affiliations:** 1 Brooklyn College, City University of New York, 2900 Bedford Ave, Brooklyn, NY 11210, USA; 2 Department of Health Sciences, College of Nursing and Health Professions, Drexel University, 3141 Chestnut St., Philadelphia, PA 19104, USA; lem324@drexel.edu (L.M.); st96d633@drexel.edu (J.D.); rms548@drexel.edu (R.S.); 3 University of Pennsylvania, 240 S 40th St., Philadelphia, PA 19104, USA; skatz2001@aol.com; 4 Monell Chemical Senses Center, 3500 Market St., Philadelphia, PA 19104, USA; pwise@monell.org

**Keywords:** ancient grains, climate change, millets, sustainability, taste, whole-grain bread

## Abstract

Millets are climate-resistant, potential alternatives to wheat that could provide environmental, food security, and health benefits (e.g., lower glycemic index). However, millets are high in phytic acid, which reduces the bioavailability of essential minerals. Millets are often fermented in Africa and parts of Asia to improve bioavailability and, thus, nutritional value, but both unfermented and fermented millets may have flavors unfamiliar to Western cultures. We conducted two pilot studies on sensory perception and liking of whole grain, United States pearl millet (*Pennisetum glaucum*), in a group of U.S. consumers. In a preliminary study, we compared pearl millet treated under five different conditions (0, 48, and 96 h of fermentation fully submerged in either distilled water or in a 5% NaCl solution at 28 °C). We found that 96 h of spontaneous fermentation in water, an inexpensive and accessible technique consistent with consumer demand for minimally processed foods, reduced phytic acid by ~72%. However, consumers (*n* = 12) rated flatbreads made with fermented pearl millet as more bitter and sour than flatbreads made with unfermented pearl millet. In a second study, participants (*n* = 30) rated liking and purchase intent for whole wheat bread with 0 to 50% (*w*/*w*) substitution of pearl millet flour. Replacing up to 20% of wheat with fermented or unfermented pearl millet had no measurable effect on liking or purchase intent. More extensive substitution compromised liking, particularly with fermented pearl millet. More work is needed, but so far, there appear to be no sensory barriers to at least partial substitution of whole-grain pearl millet for wheat in whole wheat bread for United States consumers.

## 1. Introduction

### 1.1. Millets as a Climate-Resistant Grain

Millets are important staple crop and food source for humans in many parts of the world because of its climate-resistant properties and nutrient content. Compared with wheat, millets can survive harsher growing conditions and lower rainfall in marginal lands with low fertility [1], which makes millets attractive alternative grains to be grown in the U.S., particularly in areas prone to drought. This warm-season C_4_ grass is suitable to be grown in the Western Great Plains and the Pacific Northwest regions of the U.S., with a growing season of 60 to 100 days [1]. Furthermore, compared with C_3_ grains (e.g., wheat, rice), the nutrient content of C_4_ grains (e.g., millets) remains more stable in changing climates [2]. Indeed, according to the United Nations Food and Agriculture Organization, millets production in the U.S. is on the rise, increasing from 107,240 to 376,660 metric tons between 2009 and 2019 [3]. Although an increasing number of farmers are growing this nutritious crop in the U.S., it is primarily used as animal feed [4] and has not yet achieved commercial success as an alternative grain for humans (unlike quinoa in 2013, which attracted global interest [5]). Education and information might facilitate wider acceptability of millets, but sensory properties are paramount in the acceptability of food [6]. Currently, data on consumer acceptability of millets-substituted grain products are limited, especially in North American populations [7].

### 1.2. Pearl Millet Nutrition

Pearl millet (*Pennisetum glaucum*) is the most widely grown and consumed type of millets worldwide. Pearl millet has a low to medium glycemic index according to the Glycemic Index Classification System [8] and can be an alternative gluten-free grain source for individuals with type 2 diabetes mellitus, gluten intolerance, or wheat allergy. Whole-grain pearl millet is also a rich source of minerals such as calcium, iron, and magnesium [9]. Increasing the consumption of mineral-rich whole grains may lower the risk of chronic diseases [10,11,12,13]. Overall, pearl millet is a versatile grain with numerous nutritional benefits, and exploring the potential for human consumption in the U.S. market will provide a framework for potentially introducing it as a healthy and sustainable food source.

Although consuming pearl millet in whole-grain form has greater health benefits compared with consuming refined pearl millet [10,11,12,13], pearl millet bran contains a considerable amount of phytic acid, an “antinutrient” that readily binds with positively charged minerals such as magnesium, calcium, iron, and zinc, reducing the absorption of these micronutrients [12,14]. One study reported a 40% reduction in fractional magnesium absorption when phytic acid was added to white bread [15]. This is concerning because deficiencies in some essential minerals are common in the U.S. [16,17,18,19,20], especially for individuals who consume a plant-based diet [18,21]. Although an increase in the consumption of whole-grain products is recommended by the USDA Dietary Guidelines for Americans, there is a need to identify strategies to lower phytic acid in whole grains to maximize nutritional benefits.

### 1.3. Fermentation of Pearl Millet to Improve Nutritional Benefits

There are many ways to treat grains to reduce phytic acid content, but most come with significant disadvantages. For example, decortication produces food waste and lowers essential mineral content, and thermal treatment such as popping, puffing, roasting, and blanching lowers versatility. Further, over the past 35 years, many consumers have come to prefer gentler, non-thermal-processing methods over more extensive processing [22]. Thus, the current study examined fermentation as a technique that is inexpensive, readily accessible to both consumers and businesses of all sizes, consistent with consumer desire for minimally processed foods, and effective in reducing phytic acid [23,24,25,26].

Even with a focus on fermentation, there are a variety of possible methods [23,24,25,26,27,28]. To keep the scope of this pilot study manageable, it was decided to focus on perhaps the simplest, most readily accessible method of all: soaking in water over time, i.e., spontaneous fermentation [26,27,28]. Spontaneous fermentation has long been used to treat millets in regions in which millets are staple grains and has been shown to reduce phytic acid in millets varieties grown in India and Africa [27,29]. However, to the best of our knowledge, the effect of spontaneous fermentation on phytic acid content has not been examined in U.S.-grown varieties. Another popular variation of spontaneous fermentation involves adding salt to the fermentation medium [30,31,32,33], which has been hypothesized to provide a better environment for the growth of *Lactobacillus*. Further, adding salt during fermentation to achieve a product with better sensory qualities is often advised by cookbooks (e.g., The Art of Fermentation [34]) and internet sources [35,36]. However, the effects of adding salt to the fermentation medium on mineral bioavailability warrant further investigation. Regardless, fermented foods often have a characteristic aroma that may be aversive to unacquainted consumers, and whole-grain pearl millet is known to have a slightly bitter taste and aftertaste [7,37]. These factors may compromise acceptability in a population naïve to pearl millet, but few, if any, studies have examined the effects of fermentation on the acceptability of pearl millet in U.S. consumers.

### 1.4. Current Investigation

The current pilot studies were conducted as a first step toward filling some gaps in the literature discussed above (Section 1.3). In Study 1, the effect of spontaneous fermentation on phytic acid concentration with and without salt added to the medium was investigated for a U.S.-grown strain of whole-grain pearl millet (*Pennisetum glaucum*). The effect of fermentation on the flavor of the grain, presented in simple model flatbreads, was also assessed in a small panel of U.S. consumers. Fermentation in water without added salt proved most effective in reducing phytic acid, so this fermentation technique was selected for a larger study on consumer acceptability. Study 2 assessed the effect on rated liking and purchase intent of replacing increasing amounts of both fermented and unfermented pearl millet in model whole wheat, sandwich-style bread in a group of U.S. consumers.

## 2. Study 1: Sensory Analysis of Flatbreads Made with Whole-Grain Pearl Millet

### 2.1. Rationale and Overview for Study 1

This preliminary study examined the effects of some different fermentation conditions on phytic acid concentration and sensory properties with a small group of participants. Flatbreads were used for sensory work as a model that was easy to prepare with a few additional ingredients. The main goals were to inform the selection of a single fermentation condition for a later study on consumer acceptability (see Section 3, Study 2, below) and to understand how fermentation affects flavor.

Fermentation conditions included (1) unfermented grain, (2) grain fermented in water for 48 h, (3) grain fermented in water for 96 h, (4) grain fermented in brine (5% NaCl) for 48 h, and (5) grain fermented in brine for 96 h. Five percent was chosen as representative of many culinary fermentation practices [30]. Samples were analyzed for phytic acid concentration, and participants rated the intensity of oral sensation qualities (sour, salty, savory/umami, sweet, bitter, and astringent) and liking.

### 2.2. Ethics Statement for Study 1

The protocol was reviewed by an Institutional Review Board at the University of Pennsylvania (protocol #844662) and was determined to be exempt (category 6). Participants provided written, informed consent before engaging in experimental procedures. The research was conducted according to the principles expressed in the Belmont Report and the Declaration of Helsinki.

### 2.3. Materials and Methods for Study 1

#### 2.3.1. Participants in Study 1

Twelve healthy adults with no prior training as sensory panelists, 31.1 ± 12.0 years of age, from the Greater Philadelphia area were recruited using convenience sampling methods and tested between March and May 2021. The key exclusion criterion, aside from current or chronic illness, was a history of food allergies or sensitivities for participant safety. Seven participants self-identified as men, and five self-identified as women. None were smokers.

#### 2.3.2. Stimuli for Study 1

U.S.-grown pearl millet (TifGrain 102, Kansas State University, Manhattan, KS, USA) was subjected to spontaneous fermentation for 48 and 96 h, a simple method described previously [27,38]. Whole-grain pearl millet was submerged in distilled water or salt water (brine) at a ratio of 1:2 at 28 °C under typical atmospheric pressure to encourage the growth of lactic acid bacteria present on the grain. For brine fermentation, salt (Diamond Crystal Kosher Salt, Wayzata, MN, USA) was added. The longer fermentation time was chosen based on significant reductions in phytic acid in previous work using other millets varieties [38]. After fermentation, grains were dried at 40 °C for 24 h, then milled and sieved to obtain flour. Flatbreads for the sensory test were made from 100 g of flour mixed with 50 g of water for approximately 5 min until a desired consistency was reached. The mixture was shaped into flatbread and placed on a hot griddle at medium–high heat (~200 °C) for approximately 4 min. Samples were then chilled and portioned into approximately 1 inch by 1 inch pieces. The samples were individually wrapped and labeled with random three-digit numeric codes, then packaged in a larger bag and frozen at −20 °C. Sample preparation was conducted at the Drexel University Food Lab located in Philadelphia, PA, USA. Upon enrollment, participants picked up a pack of samples in person and were instructed to freeze the pack until one hour before the study session.

#### 2.3.3. Sensory Procedures for Study 1

Participants were tested in a single session, conducted at home and supervised via video conferencing. Participants first tasted aqueous solutions of food-grade materials as exemplars of oral sensations to be rated: 2 mM citric acid (sour; Sigma-Aldrich, St. Louis, MO, USA), 120 mM sodium chloride (salty; Fisher Chemical, Pittsburg, PA, USA), 18 mM monosodium glutamate (savory or umami; Spectrum Chemical MFG Corp., New Brunswick, NJ, USA), 246 µM sucralose (sweet; Spectrum Chemical MFG Group, New Brunswick, NJ, USA), 2 µM sucrose octaacetate (bitter; Spectrum Chemical MFG Group, New Brunswick, NJ, USA), and 0.017 M pickling alum (astringency; McCormick & Company Inc., Hunt Vally, MD, USA), expectorating samples after tasting. During tasting, participants read examples of foods in which each quality is prominent. Next, participants practiced using the 100-point visual analog scales (VAS) by rating the strength of and liking for imagined and remembered sensations. Responses were entered using sliders in REDCap (Vanderbilt University, Nashville, TN, USA) with labeled endpoints (e.g., “dislike extremely” vs. “like extremely” and “not at all bitter” vs. “extremely bitter”). After a break, participants rated flatbread samples. They were instructed to chew each sample thoroughly, rate the intensity of sweet, sour, salty, bitter, savory, and astringent with the sample still in the mouth, swallow the sample, and again rate the intensity of the six qualities (aftertaste). Finally, participants rated liking. Participants rinsed their mouths with water at least twice and waited for one minute before tasting the next sample. Since preliminary tasting revealed a strong aroma, particularly for 96 h fermentation in water, participants also tasted 96 h fermented samples with their nostrils pinched shut with padded spirometry nose clips to assess the effect of aroma. Ratings for all conditions were completed in random order once, then again in random order after a break.

#### 2.3.4. Analysis of Phytic Acid

Phytic acid concentration was assessed according to the protocol of Lorenz et al. [39]. We created three batches for each of the fermentation conditions, and we assessed the phytic acid concentration for each batch in triplicates. Briefly, 0.5 g of pearl millet was mixed with 10 mL of 0.65 M HCl, left to sit overnight (~12 h), and then centrifuged at 3000 rpm for 20 min. Phytic acid dodecasodium salt (Sigma P-8810; Sigma-Aldrich, St. Louis, MO, USA) was used as the standard. Sample, standard, and blanks were added to a 96-well plate with 200 µL Wade reagent (2.5 g 5-sulfosalycyclic acid, 0.25 g iron III chloride hexahydrate, 150 mL deionized H_2_O) at room temperature for 15 min. The plate was read at 490 nm to obtain optical density (“modified Wade assay”). Concentrations were expressed as % relative to unfermented. Replicate measurements were averaged for each batch, and statistical analysis was performed across batches (*n* = 3).

#### 2.3.5. Data Analysis for Study 1

Preliminary analyses were conducted to assess retest reliability between replicate ratings using Pearson’s correlation coefficients. Mean differences between replicate ratings were also assessed using matched-pairs *t*-tests. Pearson’s correlation coefficients were used in an exploratory analysis of associations between rated attributes. Analysis of variance (ANOVA) was used to assess the effects of fermentation on the concentration of phytic acid. Repeated measures ANOVA was used to assess the effects of fermentation on rated sensory attributes, with a Greenhouse-Geisser correction of degrees of freedom for violations of sphericity [40]. Posthoc analyses were conducted using simpler ANOVAs and/or Tukey HSD tests, except for comparisons between ratings with vs. without nose clips, which were tested using matched-pairs *t*-tests (two-tailed, with a Bonferroni correction). Analyses were conducted using Statistica (Tribco, Palo Alto, CA, USA, version 13.5.0.17) and Microsoft Excel (version 16.0.6413.1000). Ratings of sensation intensity were positively skewed, so a log transform was applied before inferential analysis.

### 2.4. Results for Study 1

Only 96 h of fermentation in water significantly reduced phytic acid relative to the unfermented control but was also associated with significantly less liking, stronger bitterness, and stronger sourness. Brine fermentation produced significantly greater saltiness with liking comparable with unfermented samples but did not significantly reduce phytic acid. Comparisons between 96 h water-fermented samples rated with vs. without nose clips suggested that aroma may have contributed to ratings of some sensory attributes, including umami, bitterness, and sourness.

#### 2.4.1. Phytic Acid

**Figure 1:**
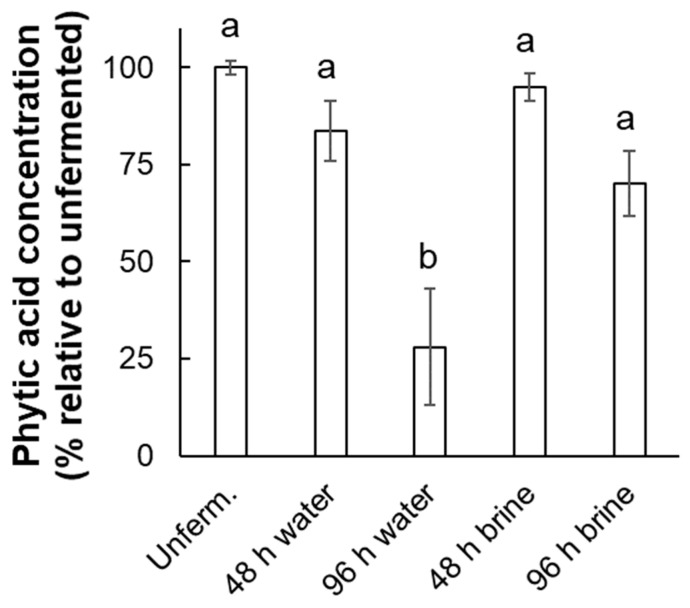
Mean (across three replicate batches, ±s.e.m.) phytic acid concentration by fermentation condition. Values are percent relative to unfermented (unfermented is 100% by definition). Lower-case letters represent statistically homogenous groups according to Tukey’s HSD posthoc test. “Unferm” = unfermented control.

Phytic acid concentration in flour varied significantly across fermentation conditions, F(4, 10) = 11.08, *p* = 0.001. A Tukey HSD posthoc analysis revealed that only the 96 h water-fermented samples contained significantly less phytic acid than the unfermented flour (Figure 1). Concentrations in brine-fermented samples were not significantly lower than in the unfermented control.

#### 2.4.2. Liking

Retest reliability was reasonable in a preliminary analysis: average r = 0.78 (S.D. = 0.12). Of the various conditions, matched-pairs *t*-tests revealed a significant mean difference only for samples made with 48 h brine-fermented grain (mean liking of 41.8 for the first rating vs. 51.6 for the replicate), and the direction of differences between replicates did not appear systematic across fermentation conditions. Thus, replicate ratings were averaged within participants before further analyses. One participant consistently rated 96 h water-fermented samples tasted without nose clips as strongly liked (mean = 95.5; the 11 other participants rated this condition at ≤20). Analyses were performed with and without this apparent outlier excluded. Mean (across replicates and participants) ratings are provided in Supplementary Table S1.

**Figure 2:**
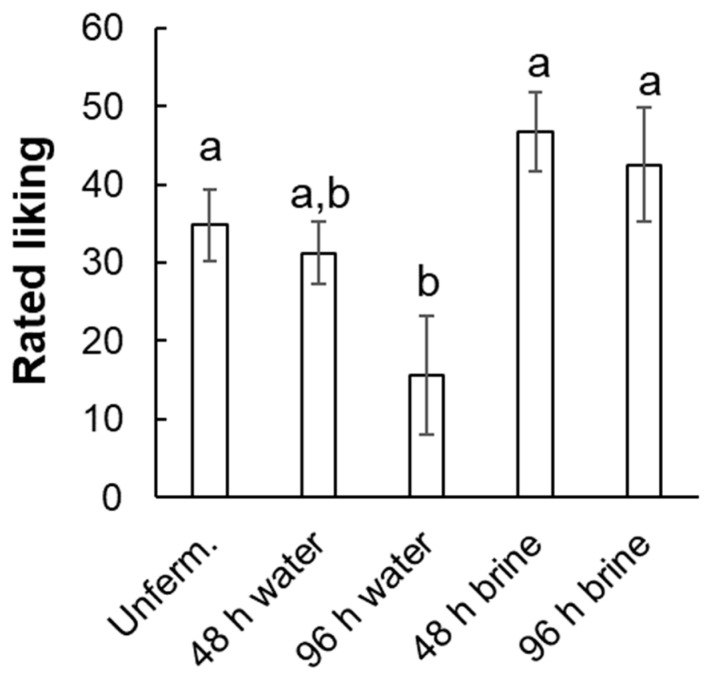
Rated liking (mean ± s.e.m.) by fermentation condition (0 = “dislike extremely, 100 = like extremely), all evaluated without nose-clips. Lower-case letters indicate statistically homogenous groups according to Tukey’s HSD posthoc test; lower-case letters indicate statistically homogenous groups. “Unferm” = unfermented control.

To assess the effect of fermentation, ratings for the five conditions without nose clips (unfermented, 48 and 96 h water-fermented, 48 and 96 h brine-fermented) were submitted to a one-way, repeated-measures ANOVA. Liking varied across fermentation conditions, F(1.9, 20.8) = 7.74, *p* < 0.003 (Figure 2). According to posthoc analysis (Tukey HSD), only flatbreads made with 96 h water-fermented grain were significantly less liked than the unfermented control. A re-analysis with the apparent outlier excluded again found a significant effect of fermentation condition, F(2.1, 21.1) = 18.10, *p* < 0.0001, and posthoc analysis (Tukey HSD) again showed that only the 96 h water-fermented samples were rated as significantly less liked than the unfermented control.

**Figure 3:**
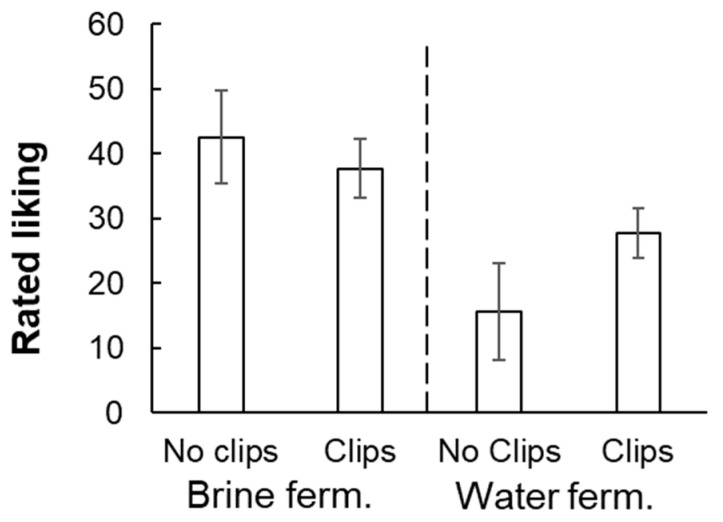
Rated liking (mean ± s.e.m.) for 96 h fermented samples, rated with vs. without nose-clips. 0 = “dislike extremely, 100 = like extremely. “Ferm” = fermented.

To assess the effects of retronasal aroma, ratings of liking for the two conditions tested with vs. without nose clips (96 h water-fermented only) were submitted to a two-way, repeated-measures ANOVA: fermentation medium (water vs. brine) X nose clips (with vs. without). The effect of the fermentation medium reached significance, F(1, 11) = 13.26, *p* < 0.004. Water-fermented samples were liked less than brine-fermented samples (Figure 3). The effect of nose clips did not reach significance (*p* = 0.40), nor did the interaction (*p* = 0.09). With the outlier excluded, effects of fermentation medium, F(1, 10) = 23.67, *p* < 0.004, nose clips, F(1, 10) = 4.97, *p* < 0.05, and the interaction, F(1, 10) = 13.71, *p* < 0.005 all reached significance. According to posthoc analysis (matched-pairs *t*-tests), 96 h water-fermented samples were significantly less liked without clips, but there was no significant difference for 96 h brine-fermented samples.

#### 2.4.3. Intensity of Oral Sensations

Retest reliability was reasonable: average r = 0.59 (S.D. = 0.24). Of the 84 combinations of rated sample, oral sensation quality, and tasting time (in mouth vs. aftertaste), matched-pairs *t*-tests yielded only two significant mean differences between replicate ratings. Thus, replicates were averaged within participants. Mean (across replicates and participants) ratings are provided in Supplementary Table S1.

Log-transformed ratings (see Section 2.3.5, Data Analysis) were first submitted to a three-way, repeated measures ANOVA: fermentation condition (unfermented, 48 and 96 h water-fermented, 48 and 96 h brine-fermented) X rating time (in mouth vs. aftertaste) X rated quality. The effect of fermentation condition reached significance, F(2.8, 31.0) = 5.00, *p* < 0.01, as did the effect of rated quality, F(3.6, 40.0) = 5.37, *p* < 0.002; and the fermentation X rated quality interaction, F(3.1, 34.4) = 5.23, *p* < 0.004. The effect of tasting time and the interactions involving tasting time did not reach significance (0.10 < *p* < 0.55).

**Figure 4:**
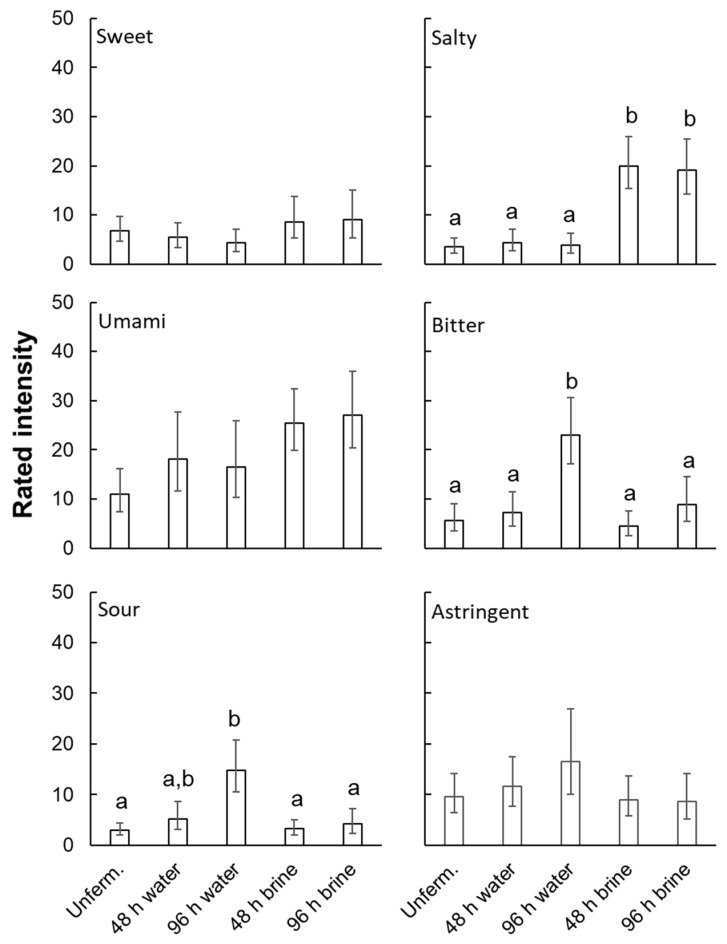
Rated intensity for each oral sensation quality (averaged across in-mouth and aftertaste) by fermentation condition (geometric mean ± s.e.m.). Lower-case letters represent statistically homogenous groups according to Tukey’s HSD test, shown only for rated attributes for which an ANOVA found a significant effect of fermentation condition. “Unferm” = unfermented.

Accordingly, data were averaged across tasting time and submitted to separate one-way ANOVAs on fermentation conditions for each rated quality. Results were significant for saltiness, F(1.5, 16.0) = 9.78, *p* < 0.004; bitterness, F(2.3, 25.1) = 8.46, *p* < 0.002; and sourness, F(2.2, 24.4) = 5.20, *p* < 0.02, but not for sweetness, umami, or astringency (0.11 < *p* < 0.32). Posthoc tests (Tukey HSD) revealed that rated saltiness was significantly greater for the two brine-fermented conditions than for other conditions (Figure 4). Bitterness was greater for 96 h water-fermented samples than for other conditions. Sourness was greater for 96 h water-fermented samples than for unfermented and brine-fermented samples, and 48 h water-fermented samples were not significantly different from other conditions.

**Figure 5:**
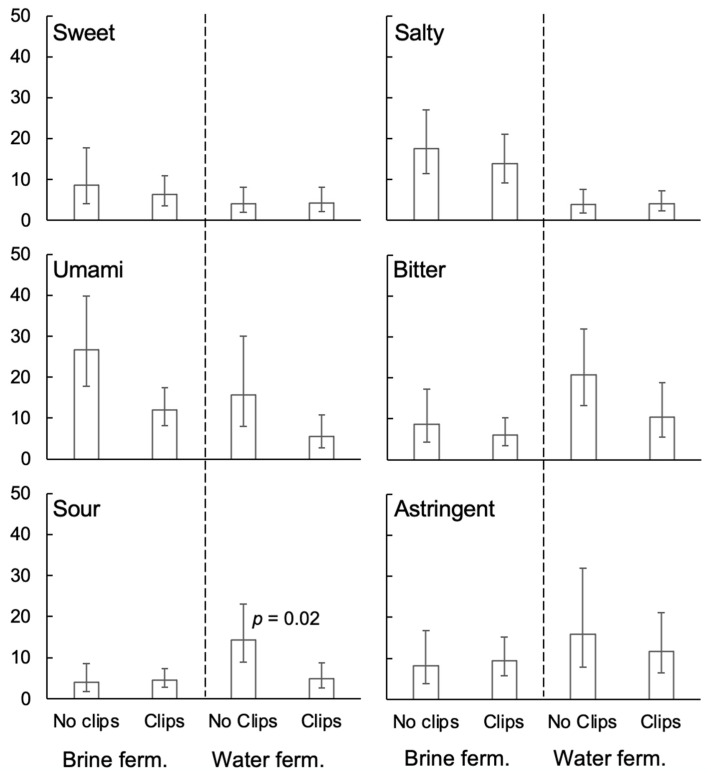
Rated intensity (averaged across in mouth and aftertaste) by oral sensation quality (separate panels) and fermentation condition (geometric mean ± s.e.m.) for 96 h fermented samples, rated with vs. without nose-clips (note: ratings without nose-clips represent the same data illustrated by the third and fifth bars in corresponding panels of Figure 4). 0 = “Not at all”, 100 = “extremely”. “Ferm” = fermented.

To assess the effects of aroma, ratings of intensity for 96 h fermentation were submitted to a three-way, repeated-measures ANOVA: fermentation medium (water vs. brine) X nose-clips (with vs. without) X rated quality. The effects of rated quality, F(3.2, 35.0) = 3.47, *p* < 0.03, nose clips, F(1, 11) = 11.77, *p* < 0.01, the rated quality X fermentation medium interaction, F(1.6, 17.4) = 6.88, *p* < 0.01, and the three-way interaction, F(2.6, 29.1) = 3.17, *p* < 0.05, reached significance. Other effects did not (0.15 < *p* < 0.38). Following up with simpler ANOVAs (fermentation medium X nose clip) yielded significant main effects of fermentation medium for saltiness, F(1, 11) = 9.77, *p* < 0.01, bitterness, F(1, 11) = 9.01, *p* < 0.02, and astringency, F(1, 11) = 7.06, *p* < 0.03. Saltiness was higher for brine fermentation, whereas bitterness and astringency were higher for water fermentation (Figure 5). The main effects of nose clips, but not the interactions, were significant for umami, F(1, 11) = 10.84, *p* < 0.01, and bitter, F(1, 11) = 5.76, *p* < 0.04, suggesting that retronasal aroma contributed to these sensations regardless of fermentation medium. For sourness, only the interaction was significant, F(1, 11) = 5.40, *p* < 0.05. According to matched-pairs *t*-tests, sourness was greater without nose clips but only for water-fermented samples, suggesting that aroma contributed to sourness only for that medium. Other effects did not reach significance (0.054 < *p* < 0.77).

## 3. Study 2—Consumer Liking of Sandwich Bread Made with Whole-Grain Pearl Millet

### 3.1. Rationale and Overview for Study 2

The model flatbreads in the preliminary study (Section 2, Study 1, above) were simple and easy to prepare but not well liked, i.e., rated below the mid-point of the scale. The flatbreads were dry and may not have been familiar to our group of U.S. consumers. A follow-up pilot study focused on liking was conducted using whole-grain sandwich bread as a model of one of the most commonly consumed, ready-to-eat whole-grain products. Varying proportions of wheat flour were substituted with pearl millet flour. The preliminary study suggested that of the few fermentation conditions assessed, only 96 h water fermentation significantly reduced the concentration of phytic acid, so this fermentation condition was included as one in which the nutrient value of whole-grain pearl millet might be improved. Bread samples were also prepared with unfermented pearl millet, both to assess how much pearl millet consumers would tolerate and for comparisons with bread samples made with fermented grain because the first study suggested that water fermentation compromised flavor.

### 3.2. Ethics Statement for Study 2

The protocol was reviewed by an Internal Review Board at the University of Pennsylvania (protocol #849824) and was determined to be exempt (category 6). Participants provided written, informed consent before engaging in experimental procedures. The research was conducted according to the principles expressed in the Belmont Report and the Declaration of Helsinki.

### 3.3. Materials and Methods for Study 2

#### 3.3.1. Participants in Study 2

Thirty healthy adults with no prior training as sensory panelists, 31.1 ± 10.0 years of age, with a body mass index of 25.9 ± 4.2 kg/m^2^ recruited using convenience sampling methods from the Greater Philadelphia area, were recruited between January and April 2021. Again, key exclusion criteria were food allergies or sensitivities and regular use of medication except birth control. Ten participants self-identified as men, and twenty participants self-identified as women. All but one of the participants self-reported that they consume bread regularly. All were non-smokers. Participants’ characteristics can be found in Table 1.

#### 3.3.2. Stimuli for Study 2

**Figure 6:**
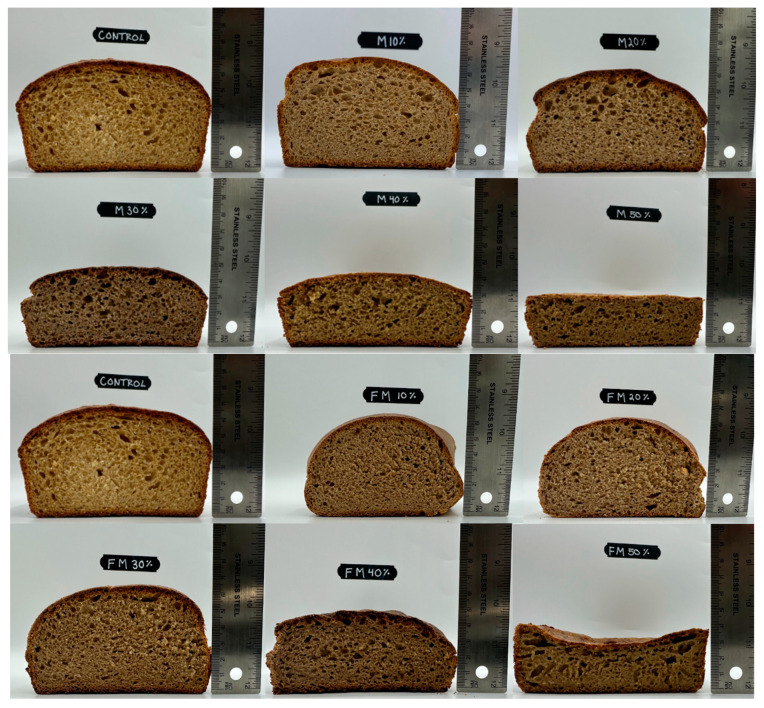
Unfermented and fermented bread models. Photos labeled with “M” = bread substituted with unfermented pearl millet flour; photos labeled with “FM” = bread substituted with fermented pearl millet flour.

The same type of pearl millet (TifGrain 102, Kansas State University, Manhattan, KS, USA) was used, both unfermented and fermented, for 96 h in water as described in Section 2.3.2 above [27]. Whole wheat flour was partially substituted with flour made with both fermented and unfermented whole-grain pearl millet in a simple bread recipe (see Supplementary Table S3). The amount of pearl millet flour ranged from barely noticeable to very discriminable according to preliminary tasting: 0%, 10%, 20%, 30%, 40%, and 50% *w*/*w* pearl millet (Figure 6). Similar to procedures in the preliminary study with flatbreads, model bread were chilled and portioned into approximately 1 inch by 1 inch pieces. Samples were individually wrapped and labeled with random numeric codes, then packaged in a larger bag and stored frozen at −20 °C. All sample preparation was conducted at the Drexel University Food Lab located in Philadelphia, PA, USA. Participants picked up study samples and were instructed to freeze samples until one hour before the study session.

#### 3.3.3. Sensory Procedures for Study 2

Participants completed four tasting sessions at home, supervised via video conferencing. In each session, participants evaluated either bread made with fermented or unfermented pearl millet flour. Half the participants were randomly assigned to one order across sessions (fermented, unfermented, fermented, unfermented), and the other half were assigned to a counterbalanced order (unfermented, fermented, unfermented, fermented). Participants received their unique sample order on a packing slip. Within each session, participants evaluated six pearl millet levels (0, 10, 20, 30, 40, and 50%) once in random order, then again in random order after a break. The participants showed the researcher the sample code on screen during the video conference prior to evaluating each sample to ensure the correct sample order. Once the sample code was confirmed, the participants examined each sample, smelled it, and then ate the sample as they normally would eat bread, though they were instructed to chew for at least several seconds to let flavor fully develop. After tasting, participants first rated overall liking, the variable of primary interest, using a 9-point hedonic scale ranging from “dislike extremely” to “like extremely.” The scale was chosen to be intuitive for participants and representative of typical methods in sensory evaluation. After rating overall liking, participants rated liking for overall appearance, color, texture, aroma, and overall flavor using the same scale. Finally, participants rated purchase intent with a three-level response: “would not buy”, “maybe buy”, and “would buy”. Responses were collected in REDCap. Participants rinsed at least twice with water after tasting each sample and waited one minute before tasting the next sample.

#### 3.3.4. Data Analysis for Study 2

Analyses addressed two main questions. First, for both bread made with fermented and unfermented pearl millet, what % pearl millet levels were significantly different from the 0% control? Second, at what % pearl millet levels did fermented and unfermented samples differ? For ratings of liking, Pearson’s correlation coefficients were used to assess retest reliability, and repeated-measures ANOVA with Tukey’s HSD posthoc tests were used to assess the effects of fermentation medium and % pearl millet.

Purchase intent was analyzed in two ways. One set of analyses treated purchase intent as an ordinal variable, i.e., a 1 to 3 rating where 1 = “not buy”, 2 = “maybe buy”, and 3 = “buy”. Effects of fermentation and % pearl millet were assessed using both repeated-measures ANOVA followed by Tukey’s HSD posthoc analyses and Friedman non-parametric tests followed by Wilcoxon tests with Bonferroni correction for multiple comparisons. Since ANOVA and the non-parametric analyses largely supported the same conclusions, only the results of ANOVA are presented below (Section 3.4.1). In another analysis, data were treated as a binary response: “buy” vs. other responses. Differences in response frequencies among conditions were first assessed using Cochran’s Q [41,42], with significant results followed by posthoc tests using pairwise McNemar tests [43]. For McNemar tests, both the (overly-conservative) exact *p* and mid-*p* (two-tailed) values were calculated in MS Excel (Version 14.0.7212.500) using published methods [44], though since the two analyses supported the same conclusions, only mid-*p* values are presented below. Tests were run on each of the four replicate responses per condition, i.e., separately for the first and second responses in both the first and second sessions. The resulting probabilities were then logit-transformed and averaged, and the average probability was back-transformed to obtain a final *p*-value. Bonferroni corrections for multiple comparisons were applied to assess which of the five % pearl millet levels were significantly different from the 100% whole-wheat control for fermented samples (*p* < 0.01) and for unfermented samples (*p* < 0.01). Comparisons between the two fermentation conditions at the six % pearl millet levels were tested at *p* < 0.0083.

### 3.4. Results for Study 2

#### 3.4.1. Liking for Model Bread

Retest reliability for ratings of liking was reasonable: average r = 0.63 (S.D. = 0.15). Of 84 combinations of rating (overall liking, liking for appearance, etc.), % pearl millet substitution, and fermentation condition, only six matched-pairs *t*-tests between replicate ratings were significant, and the direction of differences between replicates did not appear systematic. Thus, replicate ratings were averaged within participants before further analyses. Mean (across replicates and participants) ratings are provided in Supplementary Table S2. Ratings were then submitted to a three-way, repeated measures ANOVA: rated attribute (overall liking, liking for appearance, color, texture, aroma, and overall flavor) X fermentation condition (fermented vs. unfermented) X% pearl millet flour (0%, 10%, 20%, 30%, 40%, and 50%). All main effects and interactions reached significance, *p* < 0.0001 (Table 2).

**Figure 7:**
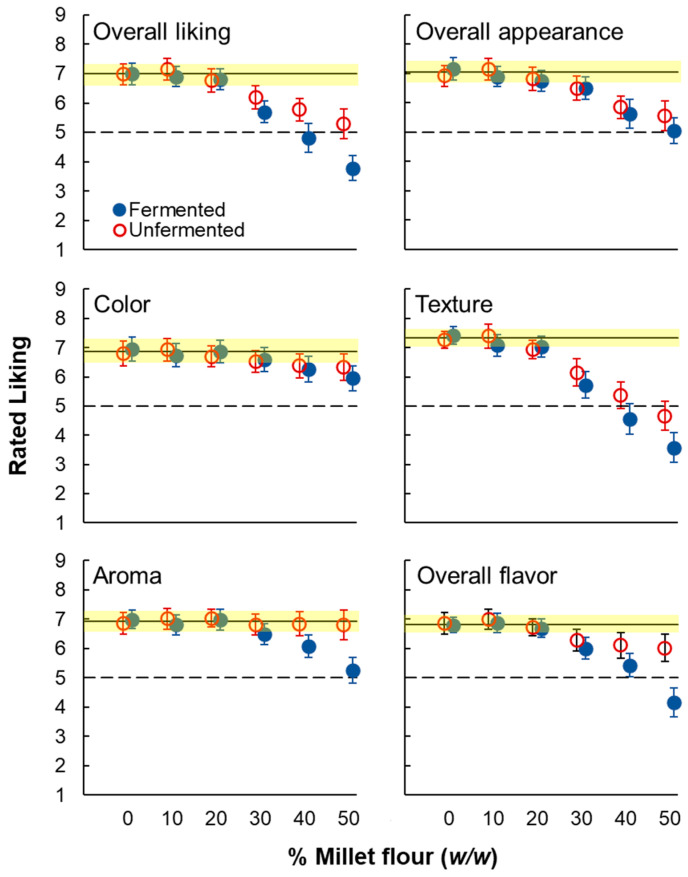
Liking (mean with 95% confidence intervals) for model whole-grain bread made by substituting increasing amounts of pearl millet flour. Red/open symbols = unfermented pearl millet, blue/filled symbols = fermented pearl millet. The solid horizontal line represents mean liking for the whole wheat control, with the yellow rectangle representing the associated 95% confidence interval. The dashed horizontal line represents the neutral point on the 9-point liking scale.

Separate two-way ANOVAs for each rated attribute revealed significant main effects of fermentation for overall liking, liking for texture, aroma, and flavor, but not for overall appearance or color (Table 3). In all four cases, bread made with fermented pearl millet was less liked than bread made with unfermented pearl millet. Further, significant main effects of % pearl millet flour were found for all six attributes: liking tended to decrease as % pearl millet increased, though the decline in liking was less marked for visual attributes, particularly for color (Figure 7). Significant interactions were found for all attributes except for overall appearance (Table 3). Inspection of mean liking by fermentation condition and % pearl millet flour (Figure 7), supported by Tukey’s HSD posthoc analyses (Table 4), showed that overall liking for 10% and 20% pearl millet samples, regardless of fermentation, was not significantly different from liking for the 0% control. Overall liking was significantly lower than control for 30, 40, and 50% pearl millet and was significantly lower for fermented than for unfermented samples for 30, 40, and 50% pearl millet (Figure 7).

Liking for overall appearance declined less markedly as % pearl millet increased, with no clear difference between fermentation conditions. Liking for color declined very little as % pearl millet increased, again with less dramatic differences between fermentation conditions. Liking for Aroma did not decline as % pearl millet increased for unfermented samples but did decline at higher levels of % pearl millet for fermented samples. Thus, the pattern of results for overall liking appears most similar to those for texture and overall flavor. Consistent with this observation, average (across % pearl millet and fermentation conditions) correlations with overall liking tended to be stronger for texture and flavor (r = 0.87 and 0.89, respectively) than for overall appearance, color, and aroma (r = 0.72, 0.70, and 0.67, respectively).

#### 3.4.2. Purchase Intent

**Figure 8:**
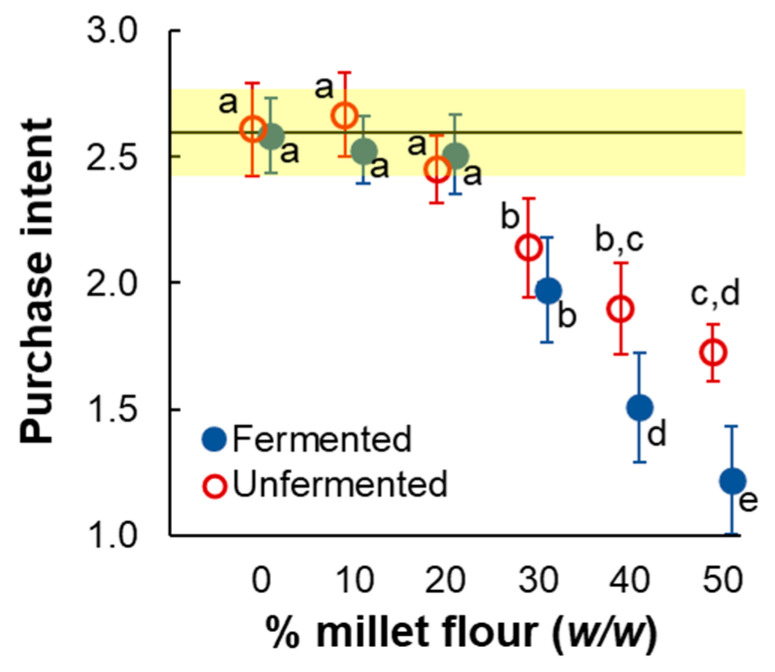
Mean (with 95% confidence intervals) strength of rated purchase intent (1 = “not buy”, 2 = “maybe buy”, 3 = “buy”) for model whole-grain bread made by substituting increasing amounts of pearl millet flour. Red/open symbols = unfermented pearl millet, blue/filled symbols = fermented pearl millet. The solid horizontal line represents mean purchase intent for the whole wheat control, with the yellow rectangle representing the associated 95% confidence interval. Lower-case letters represent statistically homogenous groups according to Tukey’s HSD test.

Treating rated purchase intent as an ordinal response produced results that closely resembled overall liking. A preliminary analysis showed that retest correlations between sessions were modest: average r = 0.46, S.D. = 0.19 (average Kendall’s Tau = 0.39, S.D. = 0.15), but statistically significant for 10 out of the 12 combinations of fermentation condition and % pearl millet. Further, differences between replicate sessions were non-significant for all 12 conditions according to both matched-pairs *t*-tests and Wilcoxon tests (0.09 < *p* < 0.99). Accordingly, data were averaged across sessions before further analysis. A two-way (fermentation condition X% pearl millet) ANOVA yielded significant main effects of fermentation, F(1, 29) = 20.57, *p* < 0.00001, and % pearl millet, F(3.3, 94.0, 29) = 88.03, *p* < 0.000001, as well as a significant interaction, F(3.8, 110.3) = 8.15, *p* < 0.0001. According to Tukey’s HSD posthoc tests, the strength of purchase intent for 10% and 20% pearl millet was not significantly different from the whole wheat control, regardless of fermentation condition. Purchase intent was significantly weaker than the control for 30, 40, and 50% pearl millet for both fermentation conditions and was significantly weaker for fermented samples than for unfermented samples for 30, 40, and 50% pearl millet (Figure 8). Non-parametric analyses supported the same conclusions.

**Figure 9:**
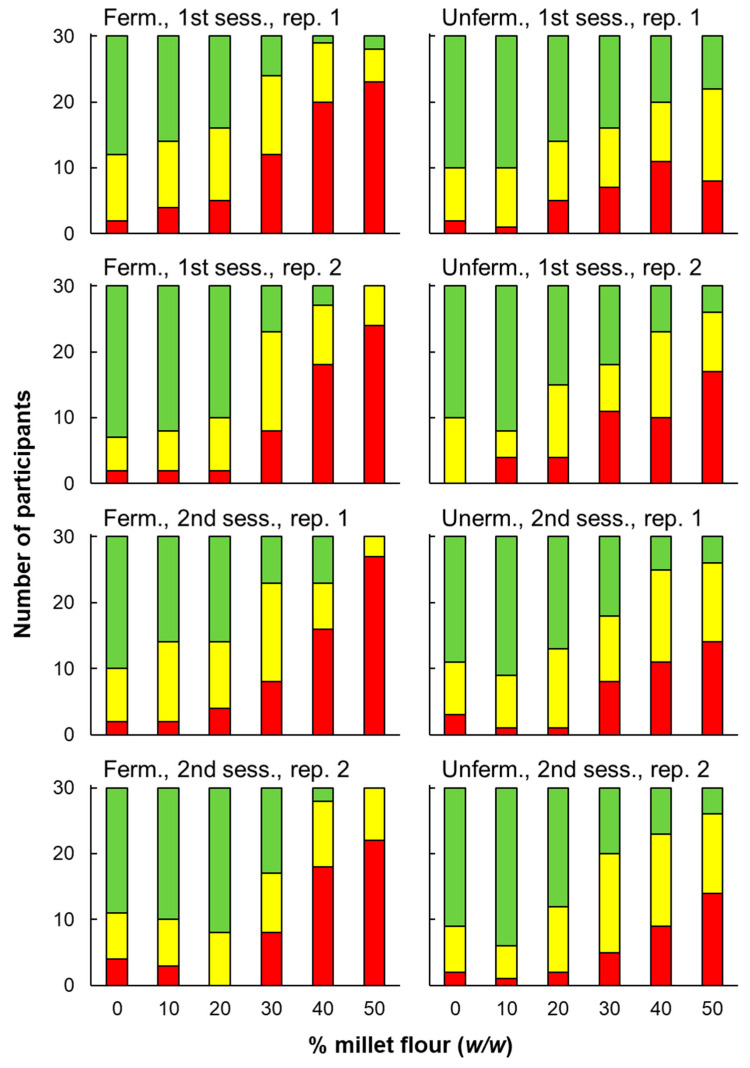
Frequencies of purchase intent responses: green bars = “buy”, yellow bars = “maybe buy”, red bars = “not buy”. “Ferm” = fermented, “Unferm” = unfermented. Responses are shown for both sessions (“sess.”) and replicate responses within sessions (“rep.”). Thus, graphs represent four replicate responses to each sample made by the same sample of 30 participants.

Treating purchase intent as a nominal response (binary: “buy” vs. others) supported similar conclusions for comparisons between pearl millet bread and the whole-wheat control but did not find significant differences between fermentation conditions. Cochran’s Q tests found significant differences among % pearl millet levels for all four replicate judgments for both fermentation conditions, *p* < 0.001 (Table 5). According to posthoc (Bonferroni-corrected McNemar) tests, neither 10 nor 20% pearl millet differed significantly from the control, regardless of fermentation condition (Table 6). Differences vs. control were significant for all higher % pearl millet flour levels for fermented pearl millet. For unfermented pearl millet, only 40 and 50% differed significantly from control, though the associated probability for 30% was low (*p* = 0.023). Regarding differences between fermentation conditions, proportions of “buy” responses did not differ significantly between fermentation conditions for any of the six levels of % pearl millet flour, two-tailed *p* = 0.52, 0.30, 0.37, 0.11, 0.05, and 0.06 for 0, 10, 20, 30, 40 and 50% pearl millet flour, respectively (Figure 9).

## 4. Discussion

To the best of our knowledge, the current pilot studies provide the first data on the potential effects on the acceptability of incorporating both unfermented and fermented pearl millet in ready-to-eat grain products in U.S. consumers. Study 1 found that of the limited number of fermentation conditions explored, only 96 h of fermentation in water significantly reduced phytic acid (by ~72%) but also made model whole-grain flatbreads more bitter and sour than flatbreads made with unfermented pearl millet. In Study 2, participants rated liking and purchase intent for whole wheat, sandwich-style bread with 0 to 50% (*w*/*w*) substitution with whole-grain pearl millet flour. Substitution with up to 20% fermented or unfermented pearl millet had no measurable effect on liking or purchase intent, but higher levels of substitution significantly compromised liking, particularly for fermented pearl millet.

### 4.1. Effect of Spontaneous Fermentation on Phytic Acid Concentration and Flavor

A preliminary analysis of phytic acid concentration was conducted to select at least one simple fermentation method likely to improve nutritional value for pilot work on consumer acceptability. Following previously published work, spontaneous fermentation in water was studied as representative of preparation methods used in areas in which pearl millet is a staple grain [27,45]. Fermentation in water reduced phytic acid by about 72% relative to unfermented grain, comparable with results for similar fermentation methods in other strains of pearl millet [38]. Forty-eight hours of water fermentation did not significantly reduce phytic acid. Brine (5% NaCl) was also tested as a medium that might be more favorable for *Lactobacilli* [46]. Adding salt when soaking beans and grains is a common culinary practice [34], but neither 48 nor 96 h fermentation in brine significantly reduced phytic acid. Salinity might have been higher than optimal and attenuated fermentation. For example, 2.5% *w*/*w* NaCl yielded the best results for sauerkraut [46]. The high salinity might also affect the reduction in phytic acid during fermentation [30,47,48].

Regarding flavor, sensory ratings from a small panel of minimally trained consumers found that the only fermentation condition that significantly reduced phytic acid also increased bitterness, increased sourness, and reduced liking in model flatbreads relative to unfermented grain. These results are broadly consistent with other work on the effect of fermentation on flavor [49,50]. Fermentation often (but not always) increases concentrations of phenolics, many of which are bitter [51,52,53]. Fermentation can also increase concentrations of amino acids and small peptides, some of which are bitter [54,55,56,57]. Of course, lactic acid bacteria fermentation increases lactic acid concentration, which can contribute to sourness [58]. Further, comparisons of ratings of 96 h fermented samples made with vs. without nose clips suggested that 96 h water fermentation imparted an aroma that may have contributed to perceived sourness and disliking. Fermentation of grains can change profiles of volatile compounds, including fatty acids [59,60], ketones [61], and esters [62], which could affect liking. To the extent an altered aroma profile in fermented foods is congruent with sourness, greater rated sourness without nose clips would be consistent with various studies showing selective enhancement of congruent tastes by aroma [63,64].

Flatbreads made with brine-fermented grain were rated as saltier than other samples, presumably reflecting higher sodium content (though sodium was not measured). Brine-fermented samples were also liked as well as samples made from unfermented grain and did not differ in bitterness. In addition to imparting a desirable salty taste, sodium tends to reduce bitterness and enhance mouthfeel [65,66,67,68]. Thus, the more favorable sensory properties may be due in part to the sensory effects of sodium. Alternatively, if smaller reductions in phytic acid for brine fermentation reflect the less extensive transformation of the grain, the better flavor might be due, in part, to lower concentrations of compounds with problematic flavor. Since some compounds with problematic flavor are important for nutritional value, it is possible that compromised flavor and increased nutritional benefit through fermentation might not be completely separable without added flavors or additional processing of the food matrix. Regardless, the preliminary study suggested that of the few fermentation conditions examined, water fermentation was most likely to significantly reduce phytic acid but also more likely to compromise acceptability.

### 4.2. Liking for Whole-Grain Bread Made with Partial Substitution by Pearl Millet Flour

The model whole wheat breads (control without pearl millet flour) were liked, with a mean score of about 7 on the 9-point hedonic scale. This hedonic score is comparable with rated liking for other standard products like popular bread or yogurt formulated with the usual amount of sugar [69,70,71]. Thus, overall liking suggested that the sandwich-style breads were more suitable models of popular products for the participants tested than the model flatbreads used in the preliminary study, which were disliked on average. Regarding the effects of pearl millet content, overall liking was not affected by substitution with 10% or 20% pearl millet flour, regardless of fermentation condition. Substitution with higher levels significantly compromised liking, with a sharper decline for fermented pearl millet. Purchase intent essentially mirrored overall liking. A more conservative, non-parametric analysis of purchase intent found no significant differences between fermented and unfermented samples in proportion to “buy” responses at any level of substitution, but observed trends were similar with greater differences between fermentation conditions at higher levels of substitution. Thus, with the caveat that ratings of liking and purchase intent were not independent since they were collected in the same trials in response to the same samples, the two measures generally supported the same conclusions.

The results of related studies conducted in Africa and the Republic of India have suggested that substitution with millets in ready-to-eat grain products or other dishes may reduce liking in some cases, but that consumer response is generally positive overall [72,73,74,75,76,77,78]. For example, in a study conducted in the Republic of Ghana, biscuits made with various mixtures of pearl millet and peanut flour were rated as significantly less liked than biscuits made with wheat flour, but all model biscuits were rated above the midpoint of the scale, i.e., in the “liked” range [72]. Further, several studies conducted in the Republic of India found that foods made with various strains of millets were not only rated in the liked range of the hedonic scales used but that some were rated as comparable with or even more liked than control recipes made with usual ingredients [74,75,76,77,78]. Consumer response may depend on both the particular strain of millets used and the dish in which it is included [78]. Regardless, the results of the current Study 2 are broadly consistent with these consumer studies in Africa and India in that all model sandwich bread made with unfermented pearl millet flour were rated on the positive side of the hedonic scale, at least as far as 50% substitution, the highest tested (Figure 7).

Fewer studies have included substitution with fermented millets, though a study conducted in the Republic of South Africa found that biscuits made with 100% fermented finger millet were less liked than biscuits made with 100% unfermented finger millet [79]. The South African study did not include biscuits with partial substitution. However, in both Study 1 and Study 2 of the current investigation, fermentation also compromised liking relative to unfermented pearl millet, at least at substitution levels of 30% or more.

There have also been few studies on consumer response to substitution with millets in populations in North America, where millets are consumed less frequently. A study conducted in Canada included refined proso millet flour in both extruded snacks and biscuits at substitution levels of 25, 75, and 100% (mixed with refined corn flour) [7]. The study did not include fermented proso millet. Regardless, as in Study 2 of the current investigation, liking tended to decrease as the proportion of pearl millet flour increased. For extruded snacks, liking was not significantly compromised relative to the 100% corn control at 25% substitution, similar to the limit of 20% pearl millet substitution without significant effects on liking observed in Study 2 of the current investigation. For biscuits, even a 25% substitution significantly compromised liking, which highlights the potential importance of the food matrix. Further, though both the Canadian study and Study 2 of the current investigation used the same 9-point liking scale, ratings of liking in the Canadian Study tended to be lower overall and on the negative side of the scale for 75 and 100% substitution. Complete or near-complete substitution gave the model extruded snacks and biscuits a bitter taste and dry texture, consistent with the model flatbreads made with 100% pearl millet used in the current Study 1. An earlier sensory study conducted in the United States on model breakfast cereals made with whole-grain proso and foxtail millet also found bitter as well as “burnt” tastes, though the earlier study included only descriptive analysis and did not measure acceptability [80]. Regardless, both current and a few past studies in North American populations suggest that extensive substitution with millets without additional adjustments to recipes might compromise liking, but that at least for some foods, limited substitution might be well tolerated even without additional ingredients or processing.

Regarding liking for specific attributes, liking for texture and overall flavor most closely resembled overall liking. Liking for appearance and color tended to decline as % pearl millet flour increased but with fewer striking differences between fermentation conditions than for flavor and texture. According to posthoc tests (Table 4), the color of unfermented samples with 50% substitution was liked significantly more than the color of corresponding fermented samples. There were no obvious differences in appearance between fermentation conditions (Figure 6). Liking for the color of some fermented samples might have been slightly lower due to scaling bias, i.e., a “horns” effect [81]. Regardless, though physical and visual properties were not formally measured, as the % pearl millet increased, bread made with both fermented and unfermented pearl millet tended to become darker and denser, with smaller crumb cells. This is generally consistent with past work on substitution with gluten-free ingredients in general and millets in particular, which may explain the general decrease in liking for color and appearance with both fermented and unfermented pearl millet flour [7,79,82,83,84]. Liking for aroma did not decrease with the percent of unfermented pearl millet flour but did decline for 30, 40, and 50% fermented pearl millet. This result is consistent with the preliminary study in which fermented samples were rated as more sour and perhaps less liked when tasted without nose clips. It suggests that aroma was an issue particular to fermented pearl millet.

### 4.3. Significance and Potential Impact

The pearl millet variety on which the current investigation focused, TifGrain 102, was developed in the United States and is suitable to be grown in drier parts of the country with minimal irrigation [85]. Further, TifGrain 102 has an expected yield of 1815 kg per acre [85], compared with an average yield of 1089 kg per acre for wheat in 2023 [86]. Thus, TifGrain 102 has the potential to be grown with good yields in more areas of the United States than wheat, allowing more sustainable local agriculture for greater food security. Further, compared with wheat and rice, the nutrient content of millets are more resistant to changes in climate [2], another way in which millets could improve food security in the face of both local and broader shifts in climate.

In addition to economic and food security benefits, research suggests that millets may also have various health benefits [87,88,89,90,91]. One frequently discussed potential health effect is improved management of diabetes [92,93,94]. Millets have lower glycemic indices compared with white rice and refined wheat [92,94], with some clinical trials demonstrating favorable effects on glycemic control [92,95,96]. Millets are dense in vital nutrients more generally, including essential minerals that are chronically under-consumed by many people in the United States and elsewhere [16,17,18,19,20,21]. Our data show the phytic acid levels in TifGrain 102 can be reduced using a simple and accessible spontaneous fermentation method, which may enhance the bioavailability of vital minerals. More work will be needed to determine whether replacing up to 20% of wheat flour with fermented pearl millet flour, a level that did not compromise sensory acceptability, is able to provide meaningful health effects. The bioavailability of minerals will ultimately depend on the participants’ individual nutrition and health status, which we did not assess in our study. How the consumption of bread formulated with fermented pearl millet may affect health outcomes in populations with mineral deficiencies could be explored in future clinical research.

Regardless of potential food security and health benefits, there must be consumer demand to make more extensive cultivation of pearl millet worthwhile [85]. Education and information may promote the consumption of pearl millet, and it is hoped that this study will help raise awareness of pearl millet as a potential alternative grain. Still, if U.S. consumers do not like pearl millet-based foods, the market will remain limited. The current investigation suggests that at least limited substitution of whole-grain pearl millet for whole wheat in one of the most widely consumed ready-to-eat whole-grain foods might be well tolerated by U.S. consumers. Thus, these pilot studies provide a foundation on which U.S. food suppliers can build in determining the viability of pearl millet-based products in the U.S. food supply and ultimately in developing products for the United States market.

### 4.4. Limitations

Our study also has several limitations. First, this pilot study included only a small subset of possible fermentation conditions. Additional manipulations of temperature, fermentation time, fermentation medium (including salinity), and use of various starter cultures might yield more favorable outcomes for flavor and nutritional value. Also, we fermented our pearl millet samples in small batches, so the feasibility of using this technique in a commercial setting is yet to be determined. Further, only one strain of U.S.-grown pearl millet was included, and even for this strain, growing location could affect the microbes present, which could, in turn, affect spontaneous fermentation. The methods used were representative of common practices that are inexpensive, consistent with minimal processing and a natural label, and easy to use for both consumers and businesses of any size but are only a starting point. Further work would also be needed to assess the effects of fermentation on other important aspects of nutritional value, such as mineral content, macronutrient content, and concentrations of amino acids and peptides.

Other limitations are associated with the group of participants, which was a convenience sample of residents of the Philadelphia area who are interested in participating in research studies. Participants may not be representative of U.S. consumers in general, as our participants tended to be younger. Further, there were more Asians and fewer Black/African American participants compared with the general population, according to the 2020 U.S. census [97]. As noted above, the proportion of pearl millet flour that could be added to model bread without significantly compromising liking was comparable with that found in similar studies in other populations. Still, caution should be used in generalizing results. The sample sizes were also limited, especially for identifying and understanding possible consumer segments. Future studies should include larger samples in different regions of the U.S., preferably with sufficient demographic representation to allow meaningful sub-group analyses.

The model foods also have limitations. The flatbreads used in the preliminary study were not well liked, though that model was not selected to be representative of U.S. foods. The model whole wheat, sandwich-style bread in the study of liking were representative of commonly consumed, ready-to-eat grain products, though the results may not generalize to other bread. For example, the addition of whole-grain pearl millet flour might be more noticeable in white bread, which tends to have a milder flavor profile, and the addition of fermented pearl millet flour might be less noticeable in sourdough bread. More generally, it would be useful to conduct studies on a wider variety of grain products.

This pilot study also did not include a manipulation of information, which is another key factor besides flavor and liking in shaping consumer acceptability [69,98,99,100]. Many consumers might accept pearl millet-substituted grain products marketed as healthful and sustainable, even if flavor or texture differences were noticeable. Further, consumers might come to accept pearl millet-substituted products with more exposure and familiarity over time, especially if changes are more gradual.

## 5. Conclusions

Consistent with past findings in other populations, the current work suggests that partial substitution of pearl millet in ready-to-eat grain products might be well tolerated by U.S. consumers. For typical whole-wheat sandwich-style bread, up to 20% by weight of either unfermented or fermented pearl millet could be incorporated with no measurable impact on liking or rated purchase intent, even with no other adjustments to the recipe. It is currently unclear if 20% substitution in bread would have meaningful health benefits. More extensive substitution may compromise acceptability, especially for fermented pearl millet, and may require additional processing or ingredients. Further, results may differ for other commonly consumed grain products like white bread. More work is needed, but thus far, there appear to be no clear sensory barriers to at least partial substitution of whole-grain pearl millet for whole-grain wheat in the U.S. food supply.

## Figures and Tables

**Table 1:** Study 2 participants’ characteristics.

Characteristic	Total *n* = 30
Age, years, mean ± SD	31.1 ± 10.0
Sex, female *n* (%)	20 (66.7)
Sex, male *n* (%)	10 (33.3)
Race, *n* (%)	
Asian/Pacific Islanders	9 (30)
Black/African American	5 (16.7)
White	13 (43.3)
Mixed	3 (10)
Body mass index, kg/m^2^, *n* (%)	
<18.5	0 (0)
18.6–24.9	14 (46.7)
25–30	11 (36.7)
>30	5 (16.7)
Bread consumption, servings/week, *n* (%)	
0	1 (3.3)
1 to 3	11 (36.7)
4 to 6	13 (43.3)
7+	5 (16.7)

**Table 2:** Results of three-way, repeated measures ANOVA on liking for whole-grain bread.

Effect	F-Ratio	DF Numerator	DF Denominator	*p*
(1) Rated attribute	13.58	3.2	91.7	<0.0001
(2) Fermentation	25.03	1.0	29.0	<0.0001
(3) % Pearl millet flour	87.71	2.1	62.3	<0.0001
1 x 2	10.62	2.7	76.9	<0.0001
1 x 3	30.63	7.7	224.0	<0.0001
2 x 3	19.80	3.1	89.5	<0.0001
1 x 2 x 3	4.49	9.3	270.9	<0.0001

**Table 3:** Results of two-way, repeated measures ANOVAs on liking for different attributes.

Attribute	Effect	F-Ratio	DF Numerator.	DF Denominator.	*p*
Overall liking	Fermentation	34.72	1.0	29.0	<0.0001
	% Pearl millet flour	93.12	2.5	72.0	<0.0001
	Interaction	17.59	3.6	104.1	<0.0001
Appearance	Fermentation	2.86	1.0	29.0	0.10
	% pearl millet flour	56.23	2.4	70.8	<0.0001
	Interaction	2.16	2.7	79.7	0.11
Color	Fermentation	0.84	1.0	29.0	0.37
	% pearl millet flour	12.63	1.8	51.8	<0.0001
	Interaction	4.73	3.4	99.0	0.003
Texture	Fermentation	23.13	1.0	29.0	<0.0001
	% pearl millet flour	121.04	2.5	73.2	<0.0001
	Interaction	9.08	3.1	89.5	<0.0001
Aroma	Fermentation	13.49	1.0	29.0	0.001
	% pearl millet flour	15.71	2.2	64.7	<0.0001
	Interaction	16.37	2.7	78.9	<0.0001
Flavor	Fermentation	28.48	1.0	29.0	<0.0001
	% pearl millet flour	42.04	2.3	66.4	<0.0001
	Interaction	23.67	3.2	93.1	<0.0001

**Table 4:** Tukey’s HSD homogenous groups (lower case letters) for statistical interactions between fermentation and % millet flour from two-way, repeated measures ANOVAs.

		% Millet Flour (*w*/*w*)					
Rating	Fermented	0	10	20	30	40	50
Overall liking	No	a	a	a	b	b,c	d
	Yes	a	a	a	c,d	e	f
Appearance	N/A *	a	a	a,b	b	c	d
Color	No	a,b,c	a,b	a,b,c,g	c,e,f,g	e,f,g	e,f
	Yes	b	a,b,c	a,b	a,c,f,g	e,h	h
Texture	No	a	a	a	b	c	d
	Yes	a	a	a	b,c	d	e
Aroma	No	a,b	a	a	a,b	a,b	a,b
	Yes	a	a,b	a,b	b,c	c	d
Flavor	No	a	a	a,b	b,c	c	c
	Yes	a	a	a,b	C	d	e

* For appearance, the interaction between Fermentation and % Millet flour was not significant (see Table 3, above). Accordingly, a Tukey HSD test was performed on the effect of % Millet flour rather than on the interaction.

**Table 5:** Results of Cochran’s Q tests of differences among pearl millet flour conditions in *p*(“buy”) responses.

	Fermented		Unfermented	
Replicate	Q, df = 5	*p* (2-Tailed)	Q, df = 5	*p* (2-Tailed)
1st session, 1st judgment	47.77	<0.000001	21.36	<0.001
1st session, 2nd judgment	74.44	<0.000001	41.43	<0.000001
2nd session, 1st judgment	46.81	<0.000001	41.81	<0.000001
2nd sessions, 2nd judgment	62.66	<0.000001	50.51	<0.000001

**Table 6:** McNemar’s mid-*p* probabilities (two-tailed) for comparisons of purchase intent for model bread made with pearl millet flour (10 to 50%) vs. 0% whole wheat control.

Comparison	Fermented	Unfermented
10% vs. 0%	0.594	0.535
20% vs. 0%	0.247	0.337
30% vs. 0%	0.001 *	0.023
40% vs. 0%	<0.001 *	<0.001 *
50% vs. 0%	<0.001 *	<0.001 *

* Statistically significant, *p* < 0.01 (see text, Section 3.3.4).

## Data Availability

Deidentified data are available upon request from the corresponding author.

## Supplementary Materials

The following supporting information can be downloaded at https://www.mdpi.com/article/10.3390/foods14050871/s1, Table S1: Ratings of oral sensation and liking for model flatbreads for all conditions averaged across replicate ratings. Table S2: Ratings of liking for model whole-grain bread for all conditions averaged across replicate ratings. Table S3: Whole grain bread base recipe.
