# Supplementary material for: Sensory Properties and Acceptability of Fermented Pearl Millet, a Climate-Resistant and Nutritious Grain, Among Consumers in the United States—A Pilot Study
Source: Foods. 2025 Mar 3;14(5):871. doi: 10.3390/foods14050871 (PMC11899355; doi:10.3390/foods14050871)
Supplement: Supplementary file 1 [file foods-14-00871-s001.zip › foods-3465157-supplementary.pdf]

Supplementary Table 1. Ratings of oral sensation and liking for model flatbreads for all conditions averaged across replicate ratings (mean ± s.e.m.)

	Brine fermentation			Water fermentation		
Unfermented	48 hours	96 hours	96 hours w/ nose-clips	48 hours	96 hours	96 hours w/ nose-clips
**Astringent**						
18.6 ± 6.7	19.5 ± 6.9	19.2 ± 6.5	17.1 ± 6.0	21.5 ± 6.2	35.6 ± 9.6	22.5 ± 6.6
**Bitter**						
12.5 ± 4.2	14.3 ± 5.7	21.2 ± 6.6	14.1 ± 5.0	14.9 ± 5.4	27.3 ± 7.2	17.2 ± 5.3
**Salty**						
7.4 ± 3.2	27.4 ± 5.7	29.5 ± 7.0	22.0 ± 6.2	10.7 ± 5.0	9.0 ± 3.8	8.1 ± 3.2
**Sour**						
5.6 ± 2.4	7.4 ± 2.8	15.0 ± 5.9*	8.2 ± 3.2	13.3 ± 5.5	20.1 ± 6.4	8.3 ± 3.6
**Sweet**						
7.8 ± 3.2	17.6 ± 5.7	20.7 ± 5.8	11.5 ± 4.7	10.9 ± 4.8	10.3 ± 4.6	10.5 ± 4.5
**Umami**						
20.7 ± 6.1	34.8 ± 6.4	37.1 ± 6.8	20.5 ± 5.8	33.4 ± 8.1	31.1 ± 8.9	13.5 ± 4.5
**Astringent Aftertaste**						
16.1 ± 6.3	18.4 ± 6.8	20.2 ± 6.5	16.2 ± 6.0	18.5 ± 5.7	34.6 ± 9.4	20.1 ± 5.6
**Bitter Aftertaste**						
11.1 ± 4.6	11.9 ± 5.0	16.4 ± 6.0	10.8 ± 4.3	15.3 ± 5.0	35.8 ± 7.3	25.1 ± 6.2
**Salty Aftertaste**						
7.3 ± 3.9	26.8 ± 5.9	24.4 ± 6.2	20.3 ± 5.7	10.3 ± 4.0	11.3 ± 4.7	8.8 ± 3.8
**Sour Aftertaste**						
4.8 ± 1.7	6.6 ± 3.1	10.5 ± 4.5	9.0 ± 3.9	14.2 ± 5.6	26.2 ± 8.4	12.3 ± 4.5
**Sweet Aftertaste**						
14.0 ± 4.6	18.4 ± 5.9	21.1 ± 6.3	14.5 ± 5.4	12.3 ± 4.7	12.9 ± 5.8	10.8 ± 5.0

Umami Aftertaste						
18.0 ± 5.2	31.5 ± 7.5	34.6 ± 7.1	16.0 ± 4.9	30.1 ± 7.3	33.0 ± 8.8	13.0 ± 4.5
Liking						
34.8 ± 5.1	46.7 ± 5.5	42.5 ± 7.6	37.7 ± 4.7	31.3 ± 4.1	15.6 ± 7.6	27.7 ± 3.9

Supplementary Table 2. Ratings of liking for model whole-grain breads for all conditions averaged across replicate ratings (mean ± s.e.m.)

Unfermented					
0% millet	10% millet	20% millet	30% millet	40% millet	50% millet
Overall					
7.0 ± 0.2	7.2 ± 0.2	6.8 ± 0.2	6.2 ± 0.3	5.8 ± 0.3	5.3 ± 0.3
Appearance					
6.9 ± 0.3	7.1 ± 0.2	6.8 ± 0.2	6.5 ± 0.2	5.9 ± 0.3	5.6 ± 0.3
Aroma					
6.9 ± 0.2	7.0 ± 0.2	7.0 ± 0.2	6.8 ± 0.2	6.8 ± 0.2	6.8 ± 0.3
Color					
6.8 ± 0.2	6.9 ± 0.2	6.7 ± 0.2	6.5 ± 0.2	6.4 ± 0.2	6.3 ± 0.2
Flavor					
6.9 ± 0.2	7.0 ± 0.2	6.7 ± 0.2	6.3 ± 0.3	6.1 ± 0.3	6.0 ± 0.3
Texture					
7.3 ± 0.2	7.4 ± 0.2	6.9 ± 0.2	6.1 ± 0.3	5.4 ± 0.3	4.7 ± 0.3

Fermented					
0% millet	10% millet	20% millet	30% millet	40% millet	50% millet
Overall					
7.0 ± 0.2	6.9 ± 0.2	6.8 ± 0.2	5.7 ± 0.3	4.8 ± 0.3	3.8 ± 0.3
Appearance					
7.2 ± 0.2	6.9 ± 0.2	6.8 ± 0.2	6.5 ± 0.3	5.6 ± 0.3	5.1 ± 0.3
Aroma					
7.0 ± 0.2	6.8 ± 0.2	7.0 ± 0.2	6.5 ± 0.3	6.1 ± 0.3	5.3 ± 0.3
Color					

7.0 ± 0.2	6.7 ± 0.2	6.9 ± 0.2	6.6 ± 0.2	6.3 ± 0.2	6.0 ± 0.3
Flavor					
6.8 ± 0.2	6.9 ± 0.2	6.7 ± 0.2	6.0 ± 0.3	5.4 ± 0.3	4.2 ± 0.3
Texture					
7.4 ± 0.2	7.1 ± 0.3	7.0 ± 0.2	5.7 ± 0.3	4.6 ± 0.3	3.6 ± 0.3

Supplementary Table 3. Whole grain bread base recipe

**Ingredients**<br>• 340g whole wheat flour, with partial millet flour substituted*<br>• 113g whole milk<br>• 152g hot water<br>• 57g melted butter<br>• 25g granulated sugar<br>• 8g salt<br>• 7g active dry yeast
**Instructions**<br>1. In a large bowl, combine all of the ingredients and stir. Transfer the dough to a mixer with dough hook attachment and knead for 10 minutes or until it begins to become smooth and supple.<br>2. Let the dough to rise until approximately doubled in bulk, ~ 1 hour at 23°C.<br>3. Deflate the dough and transfer it to a work surface. Shape the dough into an 8" log.<br>4. Place the log in an 8 1/2" x 4 1/2" loaf pan, cover the pan loosely and allow the bread to rise for about 30 minutes at 23°C, until it's domed about 1" above the edge of the pan. Bake at 177°C.

* Millet-to-wheat flour ratio ranging from barely noticeable to very discriminable
